# The Curcuminoid EF24 in Combination with TRAIL Reduces Human Renal Cancer Cell Migration by Decreasing MMP-2/MMP-9 Activity through a Reduction in H_2_O_2_

**DOI:** 10.3390/ijms24021043

**Published:** 2023-01-05

**Authors:** Verónica Ibáñez Gaspar, Tara McMorrow

**Affiliations:** UCD Centre for Toxicology, School of Biomolecular and Biomedical Science, Conway Institute, University College Dublin, 4 Dublin, Ireland

**Keywords:** curcumin, DMC, EF24, H_2_O_2_, kidney cancer, MMP, ROS, TRAIL

## Abstract

Cancer cells present high levels of oxidative stress, and although an increase in reactive oxygen species (ROS), such as H_2_O_2_, can lead to apoptosis, it can also induce cell invasion and metastasis. As the increase in ROS can lead to an increase in the expression of MMP-2 and MMP-9, thus causing the degradation of the extracellular matrix, an increase in the ROS H_2_O_2_ might have an impact on MMP-2/MMP-9 activity. The natural compound curcumin has shown some anticancer effects, although its bioavailability hinders its therapeutic potential. However, curcumin and its analogues were shown to resensitize kidney cancer cells to TNF-related apoptosis-inducing ligand (TRAIL)-induced apoptosis. This study shows that the curcuminoid EF24 in combination with TRAIL increases peroxidase activity in the renal adenocarcinoma cell line ACHN, reducing the level of intracellular H_2_O_2_ and MMP-2/MMP-9 activity, a mechanism that is also observed after treatment with curcumin and TRAIL.

## 1. Introduction

Reactive oxygen species (ROS), such as hydrogen peroxide (H_2_O_2_) or superoxide O_2_^•−^, can originate from external sources such as radiation, or be physiologically produced in the body as a by-product of cellular metabolism, mainly in the mitochondria and endoplasmic reticulum [1]. The varied, and sometimes contradictory, roles of ROS in cancer cells range from tumorigenesis to apoptosis induction due to protein and DNA damage [2,3], as in general, due to their increased metabolism, cancer cells present a higher ROS level than healthy cells [4]. Antioxidants, such as glutathione peroxidase, maintain low ROS levels by breaking down H_2_O_2_ and other reactive oxygen species in order to avoid cell damage and cell transformation [5].

ROS, in particular H_2_O_2_, have been linked to cell migration and invasion in prostate cancer cells through activation of activator protein 1 and upregulation of the heparin affin regulatory peptide gene [6,7,8,9]. In breast cancer cells, increased hydrogen peroxide levels have also been linked to increased cell migration through the FAK/Src signalling pathway [10]. In some specific breast and colon cancer cell lines, ROS production also increased the expression of matrix metalloproteinases (MMP)-2 and -9, enzymes that degrade the extracellular matrix and are involved in cell invasion and metastasis [11,12,13]. Expression of MMP-2 and MMP-9 was also specifically linked to H_2_O_2_ in lung cancer [14]. MMP-2 and MMP-9, through their enzymatic activity, facilitate cancer cell migration and therefore metastasis, and their expression has been shown to be increased in metastatic tumours [15].

TNF-related apoptosis-inducing ligand (TRAIL) is a cytokine from the TNF family that selectively induces cell death in cancer cells; however, many primary tumours are resistant to TRAIL-induced apoptosis, while others can become resistant after exposure to TRAIL. Some studies have shown that H_2_O_2_ can lead to TRAIL-induced apoptosis in TRAIL-resistant melanoma cells [16].

Natural compounds have been traditionally used in medicine to treat a wide variety of conditions, and some of them have presented varied levels of anticancer effects in vitro [17,18,19,20]. The polyphenol curcumin has been shown to inhibit cell migration and decrease MMP-2 and MMP-9 expression following H_2_O_2_ exposure in pancreatic cancer cells [21], as well as reducing MMP-2 levels in colon cancer cells [22]. Demethoxycurcumin (DMC), also naturally found in curcumin, has also previously shown a higher efficiency than curcumin to inhibit MMP-2 activation in fibrosarcoma; however, this had no impact on cell migration [23]. The synthetic curcumin analogue EF24 has been shown to increase ROS production, thus leading to apoptosis in colon cancer [24]. These compounds have previously shown anticancer effects against kidney cancer both individually but especially when used in combination with TRAIL, as they help overcome TRAIL resistance [25].

ROS might play a role in the mechanism of action of some chemotherapeutic agents. In kidney cancer cells, specifically in ACHN cells, some studies have shown the effects of anticancer drugs are linked to an increase in ROS production [26,27]. In this study we aimed to measure ROS and specifically H_2_O_2_ production in ACHN cells after treatment with curcumin and its analogues DMC and EF24, individually and in combination with the cytokine TRAIL, to analyse whether this plays a role in the mechanism of action of these drugs and their potential antimetastatic effects in ACHN.

## 2. Results

### 2.1. Curcumin, DMC and EF24 Individually and in Combination with TRAIL Reduce ACHN Cell Viability

**Figure 1:**
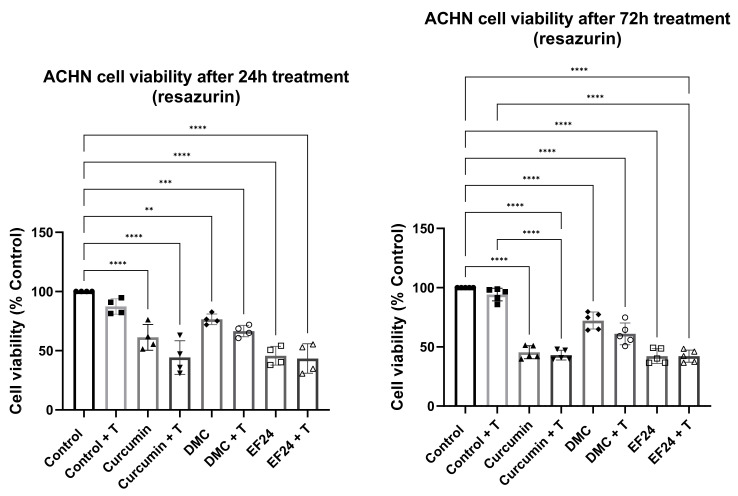
ACHN cell viability, measured through the resazurin assay, was significantly reduced throughout all treatments with IC_10_ concentrations of curcumin, DMC and EF24, individually and in combination with TRAIL. Cell viability was decreased after 24 h and further decreased after 72 h treatment, it was lowest in ACHN samples treated with EF24 (42.24%) and EF24 + TRAIL (42.17%), followed by curcumin (45.44%) and curcumin + TRAIL (43.09%) treated samples and DMC (72.19%) and DMC + TRAIL (72.19%) samples (compared to the vehicle control). 24 h *n* = 4, 72 h *n* = 5. ** *p* < 0.01, *** *p* < 0.001, **** *p* < 0.0001.

ACHN cell viability was studied using resazurin, a cell viability-indicating dye based on the metabolism of resazurin into the fluorescent resorufin. The IC10 concentrations of all treatments reduced ACHN cell viability after 24 h, which was further reduced when combined with TRAIL (see Figure 1). The lowest cell viabilities (44.25% and 43.47% compared to control) were achieved after treatment with curcumin in combination with TRAIL and EF24 with TRAIL, respectively. The reduction in cell viability was also observed after 72 h treatment (see Figure 1). Cell viability was reduced to 45.44% and 43.09% after treatment with curcumin and curcumin + TRAIL, to 72.20% and 61.02% after treatment with DMC and DMC + TRAIL and to 42.24% and 42.17% after treatment with EF24 and EF24 + TRAIL, respectively.

### 2.2. Curcuminoids DMC and EF24 in Combination with TRAIL Increase ROS Production in ACHN

**Figure 2:**
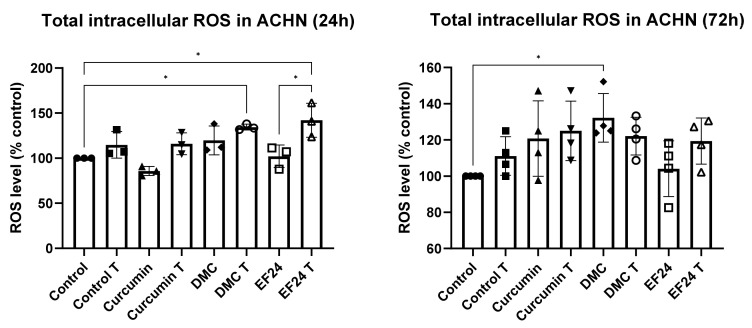
Total ROS production measured using a fluorogenic substrate, in ACHN kidney cancer cells after a 24 h or 72 h treatment with curcumin, DMC and EF24 individually and in combination with TRAIL. At 24 h, there was a significant increase in ROS production compared to the vehicle control in cells treated with DMC + T (134.44%) and cells treated with EF24 + T (142.05%). After 72 h, the increase in ROS was only significant following DMC treatment (132.22%). 24 h *n* = 3; 72 h *n* = 4. * *p* < 0.05.

Total ROS production in ACHN after treatment with curcumin and the curcuminoids DMC and EF24, individually and in combination with TRAIL, was measured using a fluorogenic substrate. After 24 h treatment, total ROS production was only significantly increased after treatment with DMC + TRAIL (134.44%) and EF24 + TRAIL (142.05%) compared to the vehicle control (see Figure 2). There was also a significant increase in ROS production when comparing EF24 and EF24 + TRAIL treatments. After 72 h treatment, although ROS was still increased throughout treatments, the increase was only significant following treatment with DMC (132.22%).

### 2.3. Curcumin and EF24 Increase Cell Death When in Combination with TRAIL

**Figure 3:**
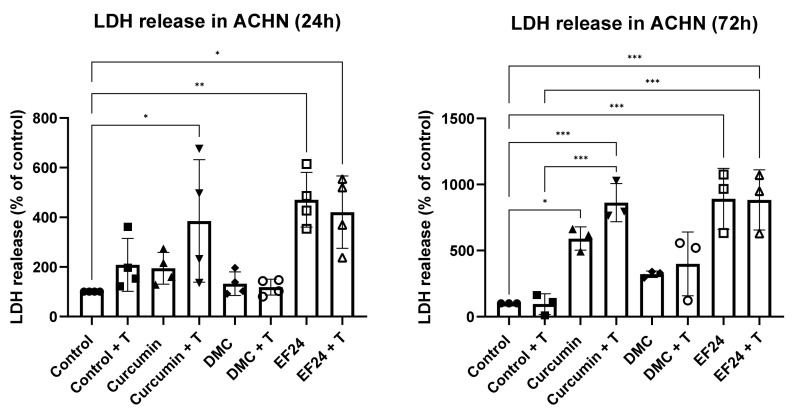
LDH release in ACHN after 24 h treatment with curcumin, DMC or EF24 individually or in combination with TRAIL. Compared to control, there was a significant increase in LDH release in cells treated with curcumin + TRAIL (385.23%) and EF24 (470.53%), both individually and in combination with TRAIL (420.59%). After 72 h treatment, all treatments increased LDH release, and was significant in cells treated with curcumin (590.87%), curcumin + TRAIL (862.89%), EF24 (891.70%) and EF24 + TRAIL (83.08%). 24 h *n* = 4; 72 h *n* = 3. * *p* < 0.05, ** *p* < 0.01, *** *p* < 0.001.

Cell death, measured by LDH release, was significantly increased in ACHNs after 24 h following treatment with curcumin (385.23%), EF24 (470.53%) and EF24 with TRAIL (420.59%) (see Figure 3). After 72 h treatment, an increase in LDH was seen in all treatments; however, it was only significant after treatment with curcumin (590.87%), curcumin + TRAIL (862.89%), EF24 (891.70%) and EF24 + TRAIL (83.08%) compared to the vehicle control.

### 2.4. Curcumin, DMC and EF24 in Combination with TRAIL Affect H_2_O_2_ Production in ACHN

**Figure 4:**
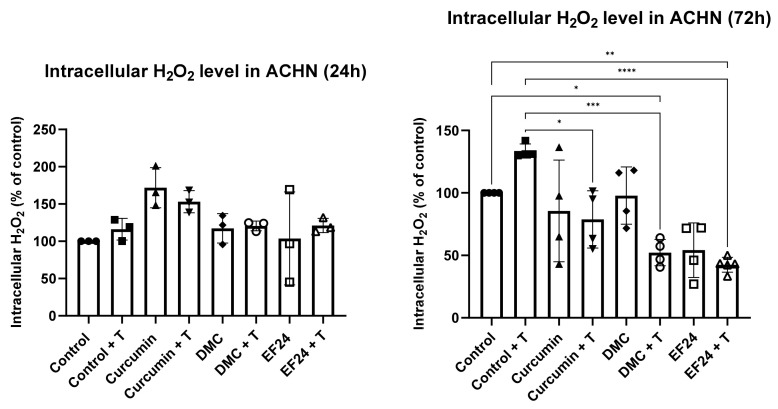
The intracellular level of H_2_O_2_ was increased after 24 h treatment with curcumin and curcumin + TRAIL; however, this increase was not significant. After 72 h treatment, intracellular H_2_O_2_ was decreased following treatment with curcumin, DMC and EF24 individually or in combination with TRAIL. This decrease was significant in DMC + T (52.18%) and EF24 + T (42.50%) treated ACHNs (compared to vehicle control), and the significance was increased when comparing the samples to the TRAIL-treated ACHNs, in which case all combination treatments showed significance. All samples were normalized to protein concentration and to vehicle control, 24 h *n* = 3; 72 h *n* = 4. * *p* < 0.05, ** *p* < 0.01, *** *p* < 0.001, **** *p* < 0.0001.

Treatment with curcumin, DMC or EF24 individually or in combination with TRAIL also had an effect on the intracellular levels of H_2_O_2_ over time. Intracellular H_2_O_2_ was initially increased after 24 h treatment in ACHN cells treated with curcumin and curcumin + TRAIL, and slightly increased in samples treated with DMC (individually and in combination with TRAIL) and EF24 + TRAIL (see Figure 4). Intracellular H_2_O_2_ was significantly reduced following 72 h treatment with curcumin + T (78.77% compared to control + T), DMC + T (52.18% compared to control) and EF24 + T (42.50% compared to control).

### 2.5. Curcumin and EF24 in Combination with TRAIL Affect Peroxidase Levels in ACHN

**Figure 5:**
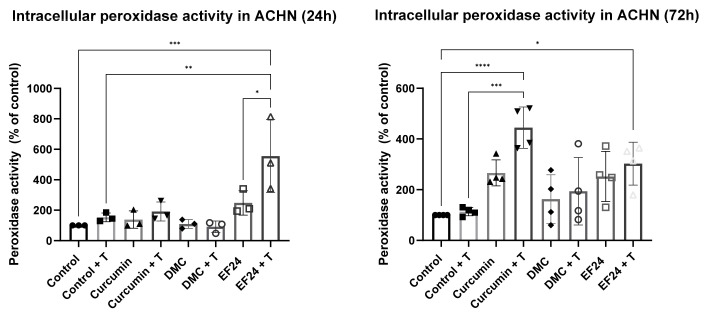
The level of intracellular peroxidase was increased in ACHN after 24 h treatment with Curcumin + TRAIL, EF24 and EF24 + TRAIL. However, the increase was only significant after treatment with EF24 in combination with TRAIL (555.51%). After 72 h, intracellular peroxidase was significantly increased after treatment with Curcumin + TRAIL (535.84%) and EF24 + TRAIL (302.52%). All samples were normalized to protein concentration and to vehicle control. 24 h *n* = 3; 72 h *n* = 4. * *p* < 0.05, ** *p* < 0.01, *** *p* < 0.001, **** *p* < 0.0001.

The level of intracellular peroxidase in ACHN cells was altered after treatment with curcumin, DMC or EFF24 individually and in combination with TRAIL. It was significantly increased after 72 h treatment with curcumin + TRAIL (535.84%) and both 24 h and 72 h treatment with EF24 + TRAIL (555.51% and 302.52%, respectively), compared to the control (see Figure 5). After treatment with DMC, both individually and in combination with TRAIL, there was a slight increase in intracellular peroxidase; however, there was no statistical significance in these changes.

### 2.6. Curcumin Treatment Individually and in Combination with TRAIL Decreases ACHN Cell Migration In Vitro

**Figure 6:**
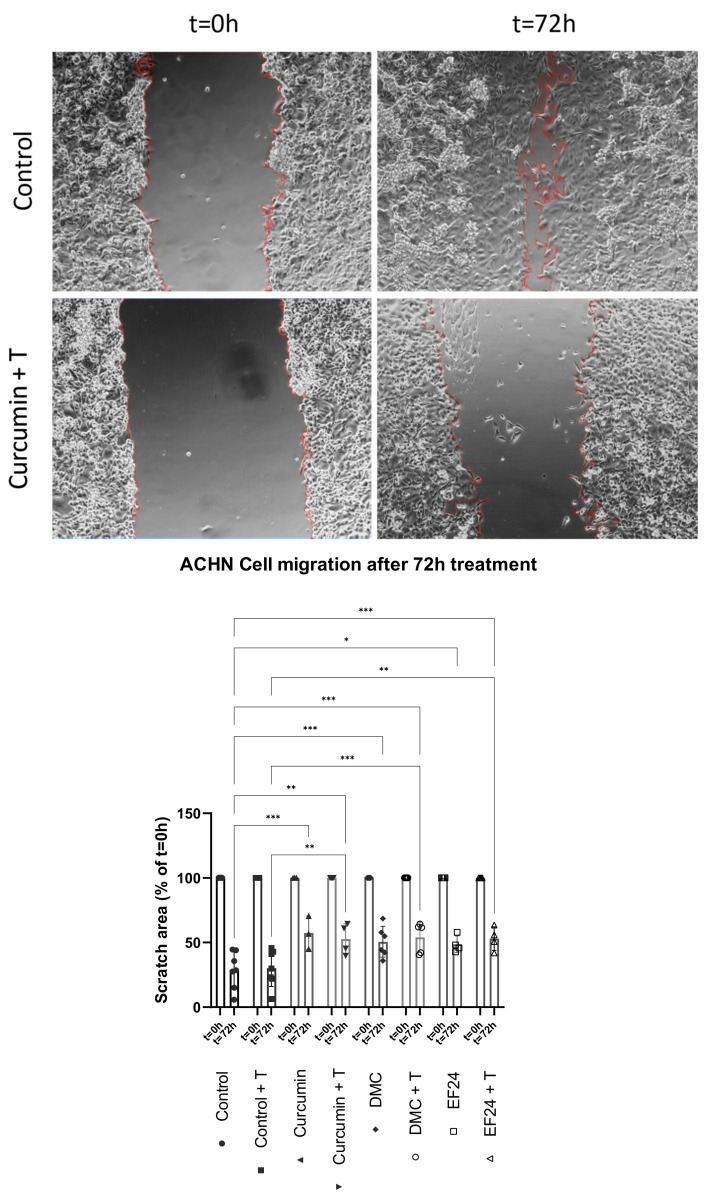
ACHN cell migration was reduced following 72 h treatment with DMC + TRAIL and EF24 + TRAIL as previously shown [18]. In this study, we show that 72 h treatment with curcumin and curcumin + TRAIL also led to a significant decrease in scratch area (52.66% compared to 28.78% in the control). Representative pictures of control and curcumin + TRAIL are shown. Red lines indicate the limits of the scratch area. (*n* = 4). * *p* < 0.05, ** *p* < 0.01, *** *p* < 0.001.

The effects of the treatments with curcumin, DMC and EF24 individually and in combination with TRAIL on ACHN cell migration were assessed using a scratch assay, in which the scratch area was measured during a period of 72 h. ACHN treatment with curcumin, DMC and EF24 seemed to reduce the migration potential of ACHN (see Figure 6), as the scratch area was increased compared to the control cells (control = 28.78%; curcumin = 57.48%; DMC = 50.63%; EF24 = 48.67% scratch area compared to t = 0 h), where cells move towards each other in order to close the gap. The addition of TRAIL further reduced the migration potential of ACHN cells when in combination with curcumin (52.66%), DMC (54.11%) and EF24 (53.05%).

### 2.7. Curcumin and EF24, Individually and Combined with TRAIL, Decrease the Activity of MMP-2/MMP-9 in ACHN

**Figure 7:**
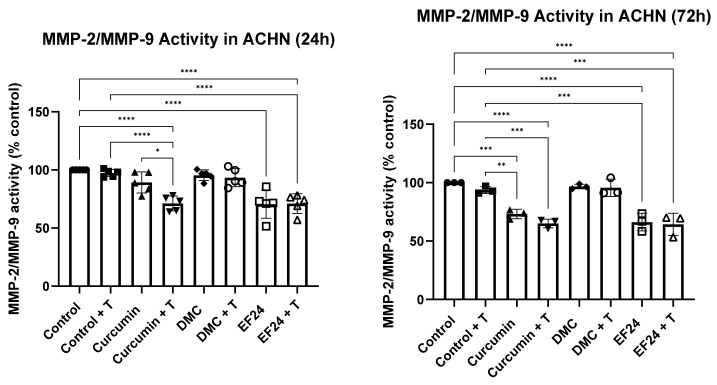
MMP-2/MMP-9 activity following 24 h and 72 h treatment of ACHN cells with curcumin, DMC or EF24 individually and in combination with TRAIL. Treatment with curcumin (89.27% at 24 h and 73.13% at 72 h) and EF24 (71.23% at 24 h and 65.04% at 72 h), both alone and in combination with TRAIL significantly reduced the activity of MMP-2 and MMP-9 (compared to control) at 24 h and 72 h, while treatment with DMC did not have an impact on the activity of these two proteins. Treatment with curcumin + T reduced the MMP-2/MMP-9 activity to 73.23% at 24 h and to 65.04% at 72 h and EF24 + T treatment reduced the MMP-2/MMP-9 activity to 66.21% at 24 h and to 64.31% at 72 h. *n* = 5 (24 h) and *n* = 3 (72 h). * *p* < 0.05, ** *p* < 0.01, *** *p* < 0.001, **** *p* < 0.0001.

The effects of curcumin and its analogues on the activity of MMP-2/MMP-9 were studied after 24 h and 72 h of treatment (see Figure 7) using a fluorogenic substrate for MMP-2/MMP-9. After 24 h treatment with curcumin and EF24, the activity of MMP-2/MMP-9 was significantly reduced to 89.27% and 70.60%, respectively (compared to the control). This effect could also be observed when ACHNs were treated with a combination of curcumin + TRAIL (71.23%) and EF24 + TRAIL (71.04%). However, no difference in MMP-2/MMP-9 activity was observed following treatment with DMC and DMC + TRAIL (95.50% and 93.33%). Longer treatment with curcumin and EF24 also led to a significant decrease in MMP-2/MMP-9 activity in ACHN (73.13% and 66.21%, respectively), which was further decreased when in combination with TRAIL. A 72 h treatment with curcumin + TRAIL of ACHN led to a decrease in MMP-2/MMP-9 activity to 65.04% and to 64.31% when treated with EF24 + TRAIL compared with the vehicle control. Treatment with DMC, either individually or in combination with TRAIL, did not significantly change the activity of MMP-2/MMP-9 compared to the vehicle control (96.77% and 95.66%, respectively).

## 3. Discussion

Curcuminoids are promising therapeutics due to their anticancer effects, especially DMC and EF24, as they present a higher bioavailability than curcumin [28,29]. Research has shown that DMC and EF24 have the potential to reduce the viability of ACHN kidney cancer cells in vitro when used in combination with TRAIL, which points to their possible effectiveness in overcoming TRAIL resistance [25,30]. In Figure 1, it is observed that the cell viability of ACHN was decreased following 24 h treatment with curcumin, DMC and EF24 individually and in combination with TRAIL. This decrease in cell viability was further enhanced after 72 h treatment; it was the most significant following treatment with EF24 (viability decreased to 42.24% compared to control) and EF24 + TRAIL (cell viability decreased to 42.17% compared to control). In this study, we demonstrate for the first time that the level of ROS in ACHN significantly increased after 24 h treatment with the combination treatment of DMC + TRAIL and EF24 + TRAIL (see Figure 2). At this 24 h time point, the percentage of cell death, as measured through LDH release, was also increased after EF24 + TRAIL (see Figure 3), which could be due to the increase in oxidative stress. However, the increase in ROS after DMC + TRAIL treatment at 24 h and after DMC treatment at 72 h did not lead to apoptosis, which points to each curcuminoid having a different mechanism of action.

The levels of H_2_O_2_ in cancer cells are higher compared to normal cells [31], and as H_2_O_2_ can act as a signaling molecule, among other functions, increased levels of H_2_O_2_ in cancer cells can lead to an increase in cancer cell migration. ACHN treatment with curcumin and curcumin + TRAIL slightly increased the intracellular level of H_2_O_2_ after 24 h; however, this increase was shown not to be significant. Interestingly, after 72 h treatment, the levels of intracellular H_2_O_2_ were drastically decreased in all treatments, and was significant in ACHN samples treated with the combination treatment of curcumin + TRAIL, DMC + TRAIL and EF24 + TRAIL (see Figure 4). Similarly, the levels of peroxidase, the enzyme responsible for breaking down intracellular H_2_O_2_, were significantly increased after 72 h treatment with the combination treatments of curcumin + TRAIL and EF24 + TRAIL (see Figure 5). These results point to the combination treatment of curcumin + TRAIL and EF24 + TRAIL leading to an increase in the intracellular levels of peroxidase in ACHN, which breaks down H_2_O_2_ and therefore decreases its intracellular levels. Although the levels of peroxidase also seemed to be increased following treatment with DMC + TRAIL, it might not be the main enzyme playing a role in H_2_O_2_ detoxification following DMC treatment in ACHN.

Kidney cancer is a highly metastatic cancer, and even after nephrectomy, metastases can develop in up to 40% of cases, which is typically associated with aggressiveness and poor outcomes [32]. Our group has showed in a previous publication that a 72 h treatment with EF24 individually and in combination with TRAIL and combination treatment with DMC + TRAIL reduced ACHN cell migration [25]. In this study, we have shown that curcumin, individually and in combination with TRAIL, also reduced the cell migration of ACHN in vitro (see Figure 6). Interestingly, the activity of MMP-2/MMP-9 was significantly reduced in cells treated with curcumin and EF24 (see Figure 7), both individually and in combination with TRAIL, which could be linked to the reduction in cell migration. MMP-2 and MMP-9 play an important role in metastasis and have been linked to lymph node metastasis in breast cancer cell lines [15] as well as to poor response to chemotherapy in osteosarcoma [33]. Further studies are necessary in order to elucidate whether other ROS, such as superoxide, might play a role in the increased cell death rate observed following some treatments, or whether other antioxidants could explain the decrease in H_2_O_2_ levels. It would also be interesting to study whether the decreased MMP-2/MMP-9 activity is due to a treatment-related downregulation in protein expression.

In conclusion, this study shows that treatment with curcumin and EF24 in combination with TRAIL increases peroxidase activity, thus lowering the level of intracellular H_2_O_2_ in the kidney cancer cell line ACHN. This reduction in H_2_O_2_ might lead to a decrease in MMP-2/MMP-9 activity, therefore inhibiting ACHN cell migration.

## 4. Materials and Methods

### 4.1. Cell Culture

The human renal cell carcinoma cell line ACHN (ATCC) was used for this study. ACHN cells were cultured in Minimum Essential Medium (Merck, Darmstadt, Germany) with 1% penicillin-streptomycin (Fisher, Dublin, Ireland) and 10% fetal bovine serum (Fisher, Ireland). Cells were stored at 37 °C and 5% CO_2_. Cells were sub-cultured at 80–85% confluency using pre-warmed trypsin (Fisher, Ireland), PBS and cell medium. For the experiments, 24-well plates were seeded at a density of 4 × 10^4^ cells/well.

### 4.2. Cell Treatments

Cells were treated with curcumin (Merck, Darmstadt, Germany), DMC (Merck, Darmstadt, Germany) and EF24 (Merck, Darmstadt, Germany) (all in DMSO) and 50 ng/mL of TRAIL (Merck, Darmstadt, Germany) was added to the wells after 4 h. The IC10 concentrations of all drugs were used (25 µM for curcumin, 10 µM for DMC and 21 µM for EF24), as previously calculated in healthy renal proximal tubule epithelial cells (RPTEC).

### 4.3. Cell Viability Measurement

After treatment, cell media were replaced with fresh media containing a 20% resazurin stock (Fisher, Ireland) solution. The plate was incubated at room temperature for 90 min on a plate rocker (covered in foil). After incubation, fluorescence was measured using the ClarioStar spectrophotometer (BMG Labtech, Ortenberg, Germany).

### 4.4. Total ROS Measurement

Prior to the beginning of the experiment, after the treatment was finished, a positive control for ROS measurement assay was set by adding 4 µL/mL of tert-butyl hydroperoxide (TBHP) to untreated cells in triplicate. The plate was incubated for 1 h at 37 °C and 5% CO_2_. Old media were then removed, and ROS fluorescent substrate (Tebu Bio, Le Perais, France) was added to each well at a final concentration of 10 µM. The plate was incubated for 30 min at 37 °C and 5% CO_2_ covered in foil. After incubation, cells were washed with HBSS buffer and fluorescence was measured using the FITC filter of a ClarioStar plate reader (BMG Labtech, Ortenberg, Germany).

### 4.5. LDH Release

LDH Release was measured using a CyQuant (ThermoFisher, Dublin, Ireland) LDH cytotoxicity kit following the manufacturer’s instructions. To summarize, after cell treatment, the cell supernatant was mixed with LDH substrate and assay buffer at a 1:1:1 ratio and incubated at room temperature for 30 min on a plate rocker covered with foil. After incubation, the same volume of stop solution as used for every other reagent was added to each well and the absorbance was measured at 490/680 nm using a Clariostar plate reader.

### 4.6. Intracellular H_2_O_2_ Measurement and Intracellular Peroxidase Activity

H_2_O_2_ release was measured using a Amplex Red Assay kit (ThermoFisher, Ireland) following the manufacturer’s instructions. Following treatment, the media were removed and cells were washed twice. Ice-cold lysis buffer (0.1% Triton X-100 in sodium phosphate buffer) was added to each well. Cells were scraped and lysates were transferred to Eppendorf tubes and vortexed every 3 min for 15 min. Cell lysates were centrifuged at 15,000× *g* and 4 °C for 15 min and the supernatants were transferred to new pre-chilled Eppendorf tubes. For the measurement of intracellular H_2_O_2_, lysates were then mixed (1:1) in a black 96-well plate with Amplex red reaction mixture, containing 10 mM Amplex Red and 0.2 U/mL HRP in sodium phosphate buffer. For measurements of intracellular peroxidase activity, lysates were mixed 1:1 with Amplex red reaction mixture containing 10 mM Amplex red and 20 mM H_2_O_2_ in sodium phosphate buffer. Plates were then incubated for 30 min at room temperature. Fluorescence was measured at 530 nm excitation and 590 nm emission. The H_2_O_2_ content and peroxidase were normalised to the protein content of each sample.

### 4.7. Cell Migration Assay

ACHN cells were cultured until a confluent monolayer was formed. Each well was scratched vertically using a p200 pipette tip. Cells were washed with PBS and treated. Images were taken using an Olympus E50 microscope and the scratch width was recorded for time point 0 h measurements. Cells were incubated at 37 °C for 72 h, at which point further images and measurements were recorded.

### 4.8. MMP-2/MMP-9 Activity

After treatment, cell media were removed and MMP-2/MMP-9 fluorogenic substrate (Cayman, Ann Arbor, Michigan) was diluted with assay to a concentration of 2 µM and added to each well. This was incubated for 1 h covered in foil at room temperature on a plate rocker. After the incubation was finished, the supernatant was transferred to a black 96-well plate and fluorescence was measured at 280 nm excitation and 360 nm emission using a Spectramax plate reader (Molecular Devices LLC, San Jose, CA, USA).

### 4.9. Statistical Analysis

Migration assay scratch area calculations and densitometry analyses were performed using imageJ Fiji. GraphPad Prism 9.0 (GraphPad Software, San Diego, CA, USA) was employed to analyze all data. MTT and Western blot data were analyzed using One-way ANOVA and a post hoc Tukey’s multiple comparison test. Migration assay data were analyzed using two-way ANOVA and a post hoc Tukey’s multiple comparison test. Results are shown as mean ± SEM. A *p*-value of 0.05 or smaller was deemed as statistically significant. Statistical significance was represented by * (*p* < 0.05), ** (*p* < 0.01), *** (*p* < 0.001), **** (*p* < 0.0001).

## 5. Conclusions

This study shows that treatment of ACHN kidney cancer cells with curcumin and EF24 in combination with the cytokine TRAIL increases peroxidase activity, thus lowering the level of intracellular H_2_O_2_. The reduction in intracellular H_2_O_2_ might then lead to a decrease in MMP-2/MMP-9 activity, therefore inhibiting ACHN cell migration. These treatments have the in vitro potential to reduce both cell viability and metastatic potential of the ACHN kidney cancer cell line.

## Data Availability

Data are available upon request from the corresponding author.
